# Identifying and Tracking Simulated Synaptic Inputs from Neuronal Firing: Insights from *In Vitro* Experiments

**DOI:** 10.1371/journal.pcbi.1004167

**Published:** 2015-03-30

**Authors:** Maxim Volgushev, Vladimir Ilin, Ian H. Stevenson

**Affiliations:** Department of Psychology, University of Connecticut, Storrs, Connecticut, United States of America; The University of Texas at Austin, UNITED STATES

## Abstract

Accurately describing synaptic interactions between neurons and how interactions change over time are key challenges for systems neuroscience. Although intracellular electrophysiology is a powerful tool for studying synaptic integration and plasticity, it is limited by the small number of neurons that can be recorded simultaneously *in vitro* and by the technical difficulty of intracellular recording *in vivo*. One way around these difficulties may be to use large-scale extracellular recording of spike trains and apply statistical methods to model and infer functional connections between neurons. These techniques have the potential to reveal large-scale connectivity structure based on the spike timing alone. However, the interpretation of functional connectivity is often approximate, since only a small fraction of presynaptic inputs are typically observed. Here we use *in vitro* current injection in layer 2/3 pyramidal neurons to validate methods for inferring functional connectivity in a setting where input to the neuron is controlled. In experiments with partially-defined input, we inject a single simulated input with known amplitude on a background of fluctuating noise. In a fully-defined input paradigm, we then control the synaptic weights and timing of many simulated presynaptic neurons. By analyzing the firing of neurons in response to these artificial inputs, we ask 1) How does functional connectivity inferred from spikes relate to simulated synaptic input? and 2) What are the limitations of connectivity inference? We find that individual current-based synaptic inputs are detectable over a broad range of amplitudes and conditions. Detectability depends on input amplitude and output firing rate, and excitatory inputs are detected more readily than inhibitory. Moreover, as we model increasing numbers of presynaptic inputs, we are able to estimate connection strengths more accurately and detect the presence of connections more quickly. These results illustrate the possibilities and outline the limits of inferring synaptic input from spikes.

## Author Summary

Synapses play a central role in neural information processing – weighting individual inputs in different ways allows neurons to perform a range of computations, and the changing of synaptic weights over time allows learning and recovery from injury. Intracellular recordings provide the most detailed view of the properties and dynamics of individual synapses, but studying many synapses simultaneously during natural behavior is not feasible with current methods. In contrast, extracellular recordings allow many neurons to be observed simultaneously, but the details of their synaptic interactions have to be inferred from spiking alone. By modeling how spikes from one neuron, statistically, affect the spiking of another neuron, statistical inference methods can reveal “functional” connections between neurons. Here we examine these methods using neuronal spiking evoked by intracellular injection of a defined artificial current that simulates input from a single presynaptic neuron or a large population of presynaptic neurons. We study how well functional connectivity methods are able to reconstruct the simulated inputs, and assess the validity and limitations of functional connectivity inference. We find that, with a sufficient amount of data, accurate inference is often possible, and can become more accurate as more of the presynaptic inputs are observed.

## Introduction

Neural computation requires fast, structured transformations from presynaptic input to postsynaptic spiking [1–3]. Changes in these transformations underlie learning, memory, and recovery from injury [4,5]. Tools for identifying synaptic weights and tracking their changes, thus, play a key role in understanding neural information processing. Traditionally, synaptic integration and plasticity are studied using intracellular recordings *in vitro*, where synaptic weights can be directly measured as the amplitude of postsynaptic potentials or currents. Although there are singular studies employing simultaneous intracellular recordings from several neurons *in vivo* [6–8], recording intracellularly from connected neurons *in vivo* is technically prohibitive. On the other hand, methods for recording extracellular spike trains are advancing at a rapid pace [9,10] and allowing the simultaneous recording of hundreds of neurons. Estimation of synaptic interactions from extracellularly recorded spike trains requires development of sensitive data analysis tools. Although strong synapses are usually readily detectable using cross-correlation analysis [11–17], where they appear as asymmetric, short latency peaks on cross-correlograms [18,19], in general, it is difficult to link the statistical relationships between spike trains to specific synaptic processes [20,21]. Here we provide empirical tests of statistical tools for such analysis using *in vitro* current injection where the true synaptic input is known.

As techniques for large-scale electrical [22] and optical [23] neural recordings continue to improve, methods for inferring interactions between the recorded neurons are needed to provide insight into the connectivity and information processing of neural circuits. Although correlational methods have long been used to study interactions between pairs of neurons [18,19], recent work has shown that statistical inference methods may be able to substantially improve our ability to detect neuronal connectivity and predict neural activity [24–26]. These model-based methods [22,27,28] are important in removing the confounds that occur with simultaneous recordings [20,29] and have revealed highly structured functional interactions, that accurately reflect the known circuit architecture, in the retina [30] and invertebrate systems [31]. However, it has proven difficult to relate functional connectivity reconstructed from spikes to the known anatomy and physiology of cortical connectivity [26,32–34]. Sparse sampling of neurons and large electrode spacing may contribute somewhat to the difficulty in interpreting the results of functional connectivity analyses of cortical circuits, but it is also unclear what information these inference methods can provide about actual synaptic inputs and what limitations there are to the use of these methods in general.

Here we examine to what extent the functional connections estimated from spike trains correspond to simulated synaptic processes in a highly controlled setting. We use *in vitro* intracellular recordings from layer 2/3 pyramidal cells in slices from rat neocortex as they respond to simulated, current-based presynaptic input. The fully-defined input is composed of excitatory and inhibitory postsynaptic currents produced by firing of large number of simulated presynaptic neurons. Since we know both the spike timing of the input presynaptic neurons and spike timing of the postsynaptic neuron, we can examine the limits of functional connectivity inference. We ask how well synaptic inputs of different amplitudes can be detected, how much data is necessary to reconstruct the amplitudes of excitatory and inhibitory synaptic inputs, and how precisely synaptic weights can be estimated from spikes alone. We briefly examine the feasibility of tracking changes in synaptic weight over time. Finally, we examine how accurately the firing of the postsynaptic neuron can be predicted from presynaptic spiking, and to what extent knowledge of multiple presynaptic inputs improves the accuracy of spike prediction.

## Results

Here we examine the relationship between simulated synaptic input and functional connections estimated from spikes using *in vitro* current injection experiments.

### Detection of artificial EPSCs immersed in fluctuating noise

**Fig 1:**
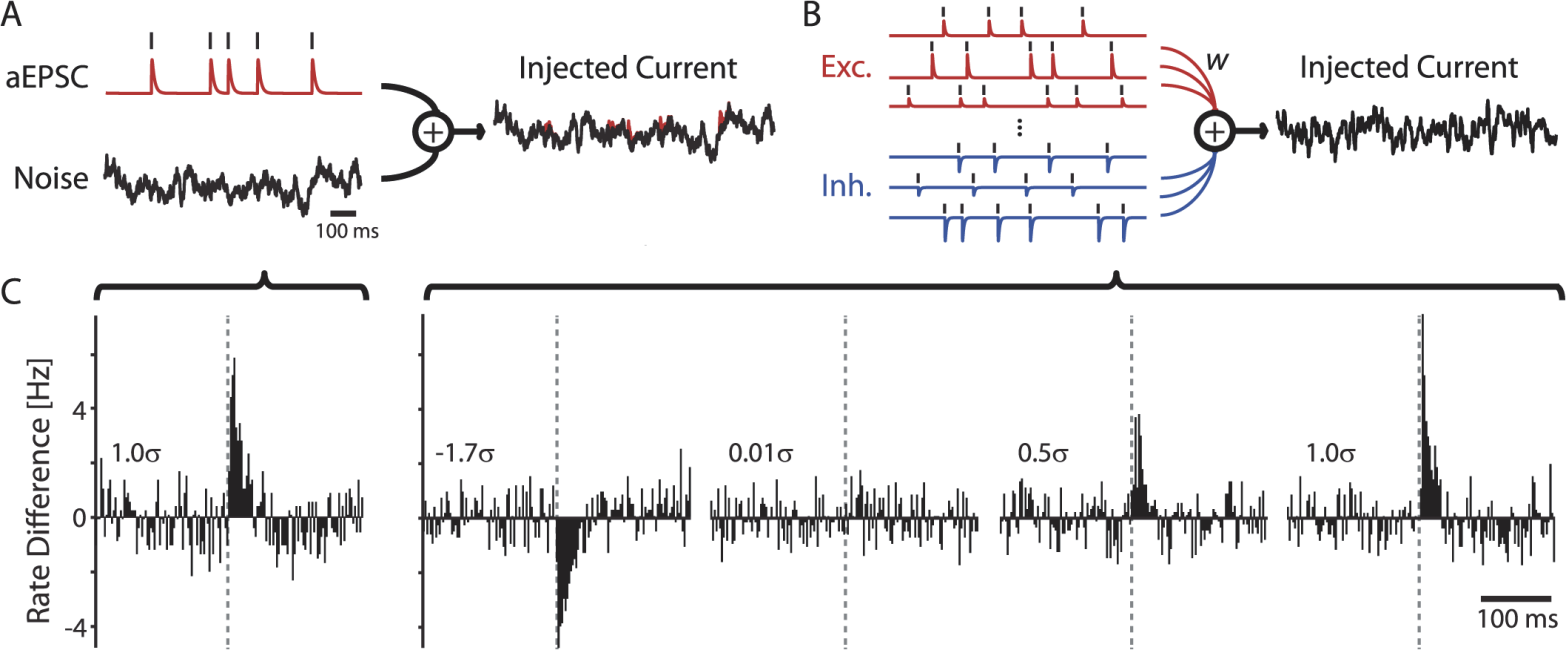
Two *in vitro* paradigms to examine detectability of neuronal connections from spike trains. A) Experimental design of a signal immersed in noise paradigm. Current for injection is composed of a repeated signal (artificial excitatory postsynaptic currents, aEPSCs, produced by simulated train of presynaptic spikes) and fluctuating noise background. B) Design of a fully-defined signal mixture paradigm. Current for injection is composed of postsynaptic currents produced by spiking of a large population of excitatory (n = 512) and inhibitory (n = 512) presynaptic model neurons firing at an average rate of 5 Hz. Different presynaptic neurons evoke postsynaptic currents of different amplitudes. Amplitude distributions of excitatory and inhibitory currents are log-normal (up to a sign change). This produces balanced excitatory and inhibitory input, and allows us to examine many (>1000) inputs simultaneously. Importantly, this large-scale approach provides a controlled framework for understanding how presynaptic inputs are reflected in postsynaptic spike statistics. C) Cross-correlations between observed postsynaptic firing and simulated spike trains from a signal immersed in noise experiment from A (left), and of 4 (of the 1024) simulated pre-synaptic neurons in fully-defined signal mixture experiment from B (right). Here and in all other figures the amplitude of input PSC is indicated in units of noise standard deviation.

In a first set of experiments the input was partially-defined. We injected into layer 2/3 pyramidal cells current consisting of three components: simulated, artificial excitatory postsynaptic current (aEPSC) from a single presynaptic neuron, fluctuating noise with standard deviation *σ*, and a DC offset (Fig. 1A). We adjusted the gain of the injected fluctuating current to produce membrane potential fluctuations with ∼15–20 mV peak to peak amplitude and DC current to achieve postsynaptic spiking ∼5Hz. After this adjustment, the overall standard deviation of the scaled fluctuating current *σ* was between 70 and 110pA. On top of this fluctuating noise, we injected aEPSCs produced by the single presynaptic input and varied the amplitude of aEPSCs in different realizations. We then analyzed the postsynaptic responses aiming 1) to examine whether input can be detected based on spikes alone, 2) to quantify how much data is necessary to detect a synaptic input of a given strength, 3) to quantify how much data is necessary to detect changes in input strength, and 4) to determine how accurately such pairwise models describe and predict spiking of the postsynaptic neuron.

Traditionally, the effects of synaptic input on postsynaptic spiking are assessed using descriptive, cross-correlation methods (Fig. 1C). Here we use a common model for estimating functional connectivity from point-process observations: a generalized linear model (GLM). We assume that postsynaptic spiking is generated by a Poisson process with a rate determined by a baseline firing rate, the recent history of the neuron’s firing, as well as input produced by presynaptic spikes (see Methods for details).

**Fig 2:**
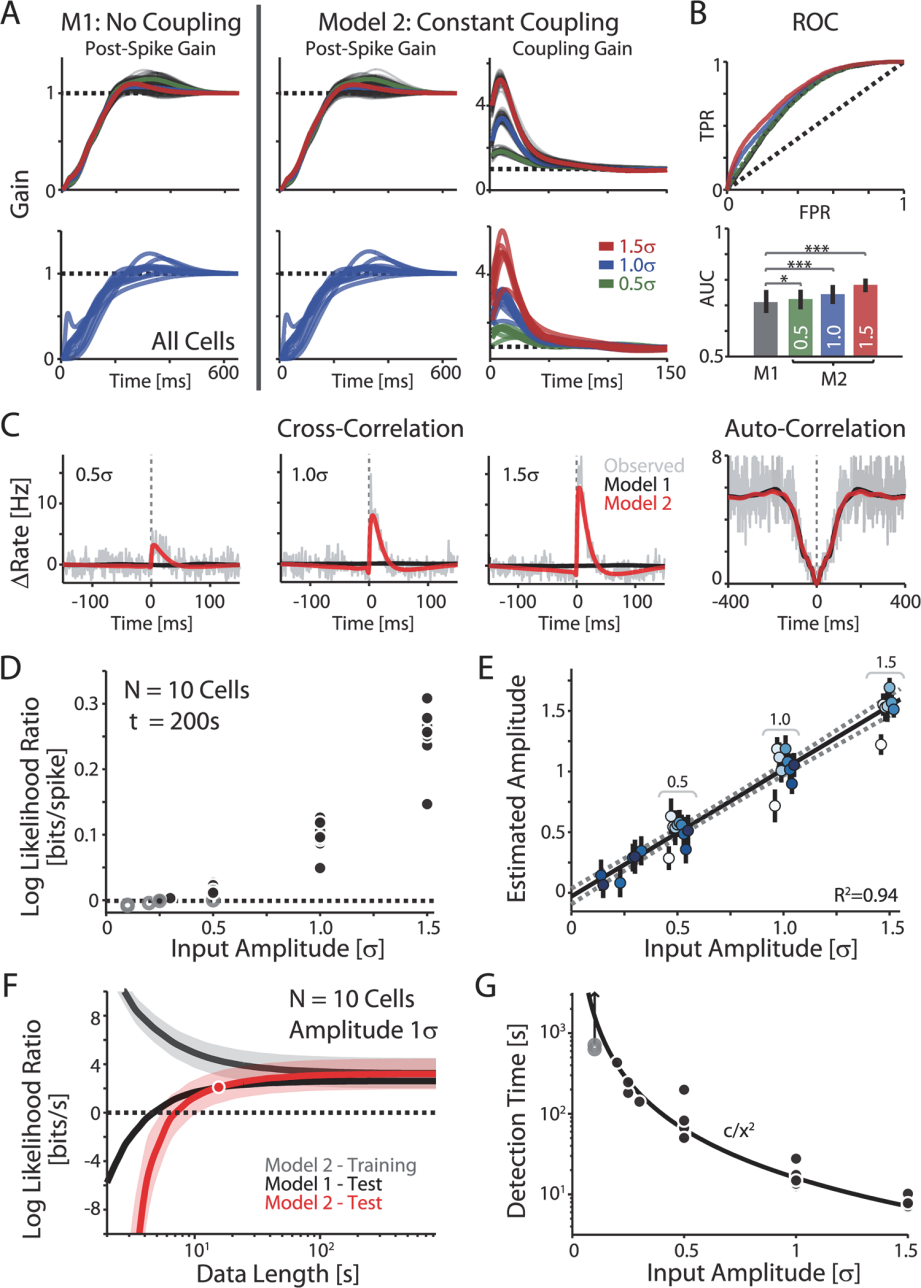
Statistical inference of synaptic connections of different strength from spike trains. A) Model fits for a typical neuron (top), and results for N = 10 cells (bottom). Model 1 (M1, No Coupling) describes only the effects of prior spikes in the postsynaptic cell on the generation of future spikes (Post-Spike Gain). Model 2 (M2, Constant Coupling) describes both the post-spike history effects (Post-Spike Gain) as well as the coupling effects following a pre-synaptic spike (Coupling Gain). Gray lines show parameters estimated from bootstrap samples; solid colored lines show their averages. B) Model accuracy: Receiver operating characteristic (ROC) curves for the example cell and area under the curve (AUC) for all cells from A. Curves show the cross-validated false positive rate (FPR) *vs* true positive rate (TPR) for spike detection in 1ms bins. Error bars denote standard deviation across cells. Note that the accuracy of Model 2, with constant-gain coupling, (M2) increases with increasing input amplitude. C) Cross- and auto-correlations for the typical cell with the model predictions. D) Detectability of synaptic connections from spike trains: Dependence of the log likelihood ratio between Models M1 and M2 on the input amplitude. Each dot represents data for one cell and one input amplitude. Black dots denote trials where M2 significantly outperforms M1 (Chi-squared test, p<0.05). E) The true amplitude of aEPSCs *vs* the estimated amplitude (coupling coefficient) using Model 2 with all data (length varies across cells 216–720s). Colors denote different cells recorded with inputs of different amplitudes, and error bars denote bootstrap standard error. F) Dependence of the log likelihood ratios of models M1 and M2 relative to a homogeneous Poisson process on the length of data used for analysis. Input amplitude of 1σ, data for N = 10 cells. Error bands denote standard deviation across cells. “*Detection time*” is defined as minimal data-length at which Model 2 with constant-gain coupling starts to out-perform the Model 1 with no connection (marked by the red point). Also note that for <50s of data the models tend to over-fit (the accuracy on training data is higher than the accuracy on test data). G) Detection time as a function of input amplitude. The dependence is well-approximated by the black curve c/x^2^. Gray dots denote recording length for those cells where no change was detected. If it exists the detection time must be longer than the recording length, as indicated by the arrow.

To determine whether an input of a certain amplitude can be “detected” given a specific set of spike trains we use the log likelihood ratio (LLR). Specifically we compare a model that predicts postsynaptic spikes based only on the recorded neuron’s spike history (Model 1) with a model that uses both spike history and presynaptic input (Model 2). These models accurately capture two different aspects of postsynaptic spiking: the spike history term captures the fact that immediately after an action potential, the probability of spike generation decreases, while the coupling terms capture the variable (excitatory, in these experiments) effect of the presynaptic input (Fig. 2A). In Model 2 the estimated presynaptic inputs correlate well with the actual amplitude of EPSCs (Fig. 2A and 2E).

Note that these effects do not correspond to exact biophysical processes, but merely capture the statistics of postsynaptic firing. The model does not aim to distinguish between the underlying processes that determine spike timing. For instance, *in vivo* results show that, although absolute refractoriness lasts only few milliseconds, generation of an action potential can influence the spike *threshold* for up to 1s [35,36]. Similarly, although postsynaptic potentials (PSPs) persist only over 10’s of ms, the effect of an EPSP on postsynaptic spike timing may last substantially longer [36,37]. Here, the estimated post-spike history and coupling effect in the models persist over 50–200ms. Both the history-only Model 1 and Model 2 with constant coupling capture statistics of the spike trains and predict spikes with reasonable accuracy. Model 2 provides an increasingly accurate prediction as the input amplitude increases (Fig. 2B). Using ROC analysis (1ms timescale, 10-fold cross-validated), we find that spikes are predicted with an area-under-the-curve (AUC) of 0.71±0.01 for Model 1, while Model 2 yields 0.72±0.01, 0.74±0.01, and 0.77±0.01 as the input amplitude increases from 0.5 *σ*, to 1.00 *σ*, to 1.5 *σ*. The relatively high accuracy of history-only Model 1 predictions reflects the fact that these neurons show substantial regularity in their ISIs (average CV = 0.54±0.02). However, Model 2 provides better spike prediction (AUC) in all cases (paired t-tests, p = 0.01, p<10^−5^, p<10^−3^). Model 1 accounts for auto-correlations fairly well, but the spike history alone cannot account for the cross-correlations between pre- and postsynaptic spikes (Fig. 2C, black lines). Model 2, using the spike history and coupling parameters together, captures both the auto- and cross-correlations present in the observed spiking (Fig. 2C, red lines). Consistent with results of cross-correlation and area-under-the-curve analysis, the model with coupling provides a better fit to the data than the spike-history alone model when the amplitude of added synaptic input is 0.5 *σ* or larger. For a fixed amount of data (recording length 200s), modeling a synaptic input with amplitude < 0.5σ does not improve prediction of the postsynaptic spikes over a spike history-only model (Fig. 2D). Nevertheless, even for these weaker inputs (0.1 *σ*, 0.2 *σ*, 0.25 *σ*, and 0.3 *σ*) the model parameters are able to accurately reproduce the relative amplitude of the presynaptic input (Fig. 2E). Here we use the kernel mean over the first 25ms to summarize the coupling strength in Model 2 (see Methods).

Detectability of synaptic connections from spike trains depends strongly on how much data is available. With only a short recording of pre- and postsynaptic spikes it is difficult to determine if two neurons are “connected”. Both Model 1 with spike history-only and Model 2 with coupling tend to over-fit data from recordings shorter than ∼5–10s (LLR<0 when compared to a homogeneous Poisson model which describes only the baseline firing; Fig. 2F). As the amount of available data increases over-fitting is reduced, and, with sufficient data, Model 2 is more accurate than Model 1 if there is truly a synaptic connection between the neurons. We define *detection time* as the length of data at which Model 2 with coupling provides a better fit than Model 1 with history only (Fig. 2F, red point). For the data in Fig. 2F, where the pre- and postsynaptic neurons each have 5Hz firing rates and the amplitude of the synaptic connection is 1σ, this crossing point occurs around 20s. By varying the input amplitude we find that the recording time needed to detect an input of amplitude *x* falls off as approximately *c* / *x* ^2^ (Fig. 2G), with c = 16.0±0.1s.

**Fig 3:**
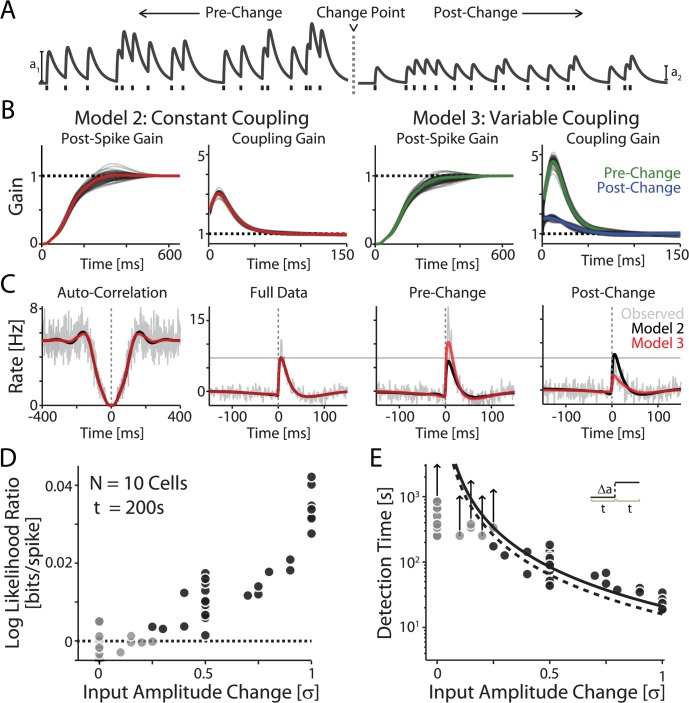
Statistical inference of changes in input strength. A) Example traces of aEPSCs before and after an amplitude change. In experiments, these traces were immersed in fluctuating noise current. B) Model fits of a data set consisting of two recordings from the same cell but with different amplitudes of aEPSCs (1.5σ and 0.5σ). Model 2 (M2, Constant Coupling), captures the effect of postsynaptic spike history and coupling to a presynaptic input on the full data. However, to detect changes in input strength these parameters must change over time. In Model 3 (M3, Variable Coupling), we separate the coupling effect into one regime before the change-point and one after the change-point. The post-spike history is the same before and after. C) Auto- and cross-correlations for the full data (left), and cross-correlations for the pre-change and post-change portions. Note that while Model 2 and Model 3 both describe the full data well, only Model 3 with variable-gain coupling describes separated pre-change and post- change portions. For comparison, the horizontal gray line denotes the peak of the cross-correlation when the full data is used. D) Log likelihood ratio between Models 2 and 3 for various amplitude changes (200s of data for all cells/changes). Black dots denote cases where Model 3, with variable-gain coupling, provided significantly better fits than Model 2, with constant-gain coupling (Chi-square test, p<0.05). E) Recording time necessary to detect changes in input amplitude. The dashed curve shows the detection times for constant-amplitude inputs (from Fig. 2G). Note that only slightly more data is necessary to detect changes in amplitude than to detect an input. Gray dots denote recording length for those cells where no change was detected. If these inputs are detectable the detection time must be longer than the recording length, as indicated by the arrows.

In addition to detecting whether two neurons are connected or not, for studying plasticity, it is important to determine whether the strength of a connection changes, for instance, after applying an experimental manipulation, such as high frequency stimulation or a conditioning session. To study the detectability of a change in connection strength, we constructed artificial data sets from the recorded data, in which the strength of the synaptic connection between two neurons changes at time point t, from amplitude **a**_**1,**_ during time period from 0 to t, to amplitude **a**_**2,**_ for time t to 2t (Fig. 3A). As in previous analysis, we use the likelihood ratio to determine whether the synaptic weight has changed. We compare Model 2, used previously, with a single synaptic input of a constant amplitude throughout the recording time (0 to 2t) to a new Model 3 which allows the synaptic input to differ before and after time t (Fig. 3B).

As expected, both Models 2 and 3 accurately account for the auto-correlation statistics and the cross-correlations between pre- and postsynaptic spiking on the full data (Fig. 3C, left). However, only Model 3 can accurately track changes of firing statistics produced by changes of synaptic strength and accurately describe the cross-correlation before and after the change-point t (Fig. 3C, right). As in the case of detecting a connection, the parameters of Model 3 accurately reproduce the amplitude changes in the presynaptic input (R^2^ = 0.96±0.01, data not shown).

Detectability of synaptic weight changes in long recordings is comparable to the detectability of connections of constant strength. In 200s long recordings, a likelihood ratio test between Models 2 with constant coupling and Model 3 with variable coupling reveals the change only for Δ*a* > 0.25σ (Fig. 3D, compare to Fig. 2D). Again, the ability to detect a change strongly depends on the recording length. With less data available, detecting changes in synaptic strength becomes more difficult than simply detecting a connection. The recording length necessary to detect a change *x* = Δ*a* of connection strength after a known change-point falls off as approximately *c* / *x* ^2^ with c = 21.5±0.1s (Fig. 3E).

### Detection of connectivity in fully-defined input setting

In a second experimental protocol, rather than injecting aEPSCs from a single synaptic input immersed in noise, we inject current that is produced by the activity of a *large number* (N = 1024) of spiking presynaptic neurons (Fig. 1B). We used a presynaptic population consisting of the equal number of excitatory and inhibitory neurons, with log-normal distribution of synaptic amplitudes (same distribution, positive weights for excitatory, negative weights for inhibitory), and PSC kernels consisted of the same difference of two exponentials with time constants of 0.5ms and 5ms. The average input had an amplitude of 0.15 σ (corresponding to ∼15pA, depending on σ), and membrane potential responses to injection of this current again mimicked the statistics of membrane potential fluctuations *in vivo* with amplitudes of 15–20mV [8,38,39]. This paradigm for injection of fully-defined current allows us to examine the detectability of excitatory and inhibitory inputs of multiple amplitudes using the same recording [40]. Using this paradigm we examined 5 additional cells, 3 of which were driven to fire at ∼5Hz, and the other 2 were each driven at several different rates: ∼1Hz, ∼5Hz, and ∼10Hz (produced by varying the DC offset). We then analyze the postsynaptic responses using the same pair-wise model comparison techniques as we used for analyzing single-input experiments.

**Fig 4:**
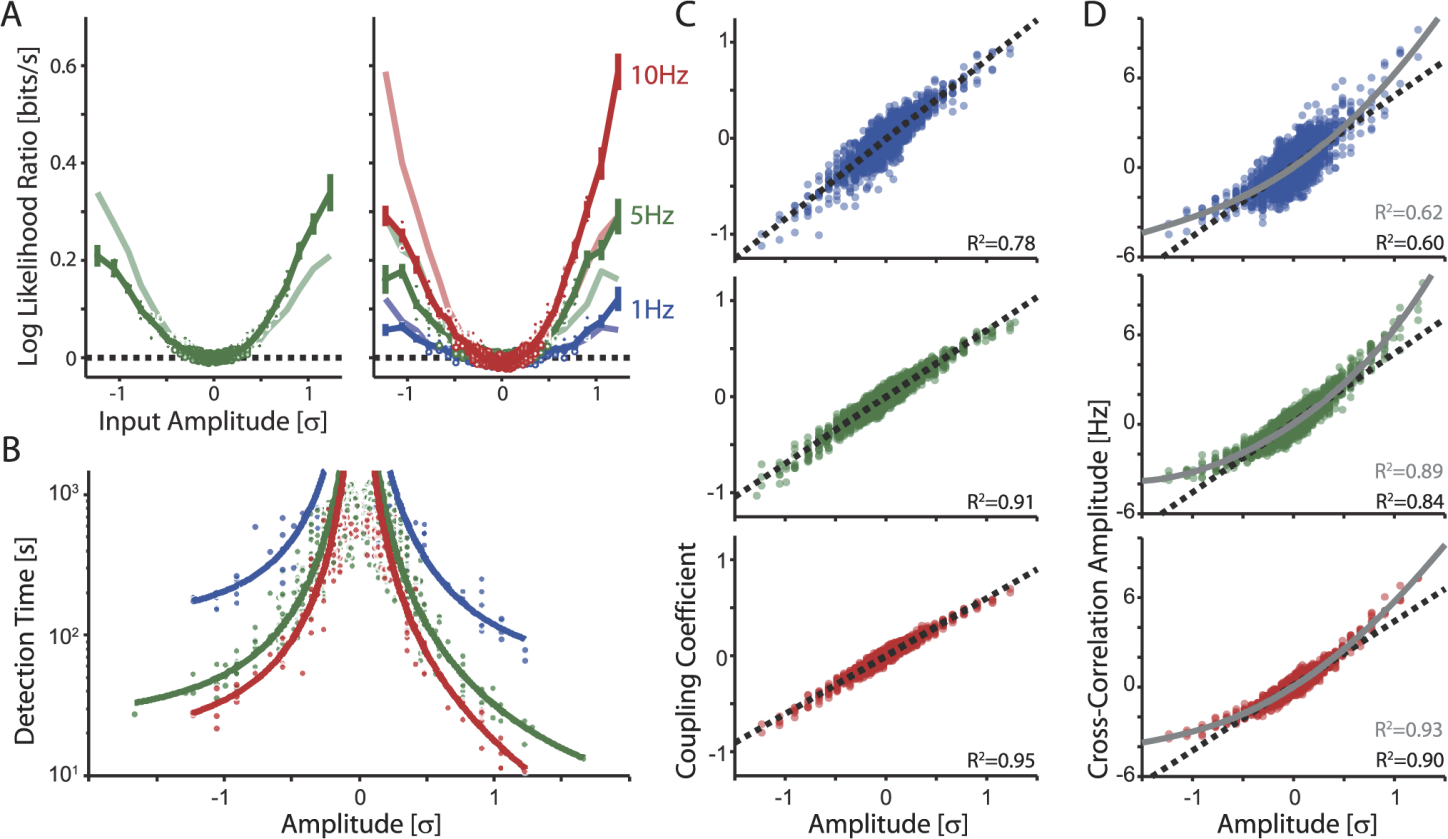
Detection of individual synaptic inputs from responses to injection of fully-defined signal mixture. Injected current is composed of inputs of different strength from a spiking population of excitatory and inhibitory presynaptic neurons, as described in Fig. 1B. This allows us to examine multiple amplitudes simultaneously. A) Detectability of synaptic inputs of different strengths: Dependence of the log likelihood ratio between models with no coupling (M1) and with constant-gain coupling (M2) for each of the 1024 inputs which composed the injected current. Left plot: data from a layer 2/3 pyramidal cell, recorded with firing rate ∼5Hz. Right plot: data from a different layer 2/3 pyramid, with postsynaptic firing rate maintained at ∼1Hz, ∼5Hz, and ∼10Hz, as indicated. Filled dots denote cases where Model 2 with coupling, provided significantly better fits than Model 1 with no coupling (Chi-square test, p<0.05). Error bars denote SEM. As in experiments from the signal immersed in noise paradigm, stronger inputs are detected more accurately. Note that detection of small-amplitude inputs improves with higher firing rates (filled symbols at lower amplitudes). However, amplitudes less than ∼0.25σ were not detectable even at 10 Hz. Note a substantial asymmetry in detectability between excitatory and inhibitory inputs. To illustrate the more reliable detection of excitatory inputs, lighter colors show the average log-likelihood ratio curves reflected about input amplitude 0. B) Detection time for inputs of different amplitudes (data for N = 4 cells). As in experiments with signal immersed in noise paradigm, detection time drops as ∼c/x^2^. Note that detection of inhibitory inputs requires longer recording periods than detection of excitatory. C) Ground truth PSC amplitude *vs* the estimated coupling coefficient for 1Hz (top), 5Hz (middle), and 10Hz (bottom) conditions (N = 4 cells for the 5Hz condition, N = 2 cells for the 1Hz and 10Hz conditions, >800s of data in all cases). Estimated coefficients accurately reconstruct the true amplitudes, with the accuracy improving with increasing spike rate. Dashed lines denote a linear fit. D) Ground truth PSC amplitude *vs* the amplitude of the cross-correlation (25ms window following pre-synaptic spikes). The cross-correlations also reflect the true amplitudes, but nonlinearly. Gray curves denote a 4^th^ order polynomial fit.

Similar to results from the single input experiments, analysis of the fully-defined input experiments shows that the larger an input is the larger an effect it has on postsynaptic spike prediction, and the more readily it is detected. High-throughput experiments with injection of the fully-defined input allowed us to reveal further features of input detection. The detectability is influenced not only by amplitude, but also by the sign of the input (excitatory *vs* inhibitory) and the postsynaptic spike rates (Fig. 4A). Inhibitory inputs are detected less readily than excitatory. They have less impact on the postsynaptic firing, and thus are less accurate in predicting output spikes compared to excitatory inputs of the same magnitude (the log likelihood ratios comparing Model 2 with coupling to Model 1 with spike-history alone are 58±2% smaller for inhibitory inputs). As in single-input experiments, detection time decreases with amplitude approximately as *c* / *x* ^2^. With these data we find c = 111, 32, and 18s for detection of excitatory inputs at 1Hz, 5Hz, and 10Hz output rates and c = 182, 46, and 29s for detection of inhibitory inputs at these rates. Across postsynaptic firing rates *r*, these times are well approximated by *c* / *rx* ^2^ (Fig. 4B). On average, inhibitory inputs required 32±3% more data for detection than excitatory inputs. Although detection time likely depends also on presynaptic firing rates as well as time course of PSCs (not just their amplitude), here, for making the comparison clear, presynaptic rates for all inputs were held at 5Hz and PSC kernels had the same time course, differing only in amplitude.

In the fully-defined input setting, we can examine, in a single cell, how accurately model estimates of synaptic weights (of different amplitude and sign) capture the actual values. The coupling coefficients accurately reconstruct both excitatory and inhibitory input amplitudes over a broad range, and this reconstruction becomes more accurate with higher postsynaptic firing rates (Fig. 4C). The pair-wise cross-correlations (0–25ms following presynaptic spikes) also reflect the input amplitudes fairly well but are much more nonlinear (Fig. 4D). Uncorrected cross-correlations tend to underestimate the relative amplitude of strong inhibitory inputs, but overestimate the relative amplitude of strong excitatory inputs.

Next, we ask how modeling multiple inputs simultaneously might affect the detection of individual inputs. Model 2, with post-spike history and coupling to a single input, can be extended to capture multiple presynaptic inputs simply by adding extra coupling parameters for each additional input. However, modelling all 1024 inputs in detail is computationally prohibitive. Here we consider two alternatives: 1) a model with 64 coupling terms where inputs of the same amplitude are grouped together, and 2) a bilinear model where the 1024 inputs are restricted to have the same shape. Since the presynaptic inputs in our experiments were specifically grouped together and all have the same PSC shape, these alternative models both provide accurate descriptions of the data (see Methods). It is important to note, though, that these models are simplifications. In typical multi-electrode spike recordings grouping information would not be available, and PSCs at different synaptic connections would have different shapes.

**Fig 5:**
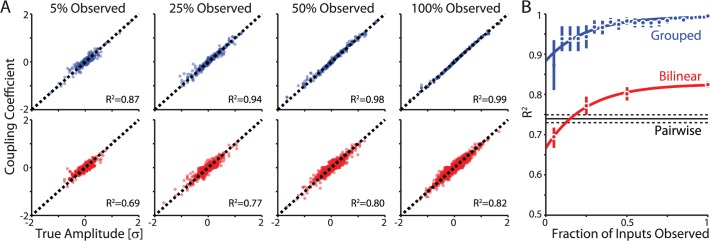
Coupling estimates become more accurate with increasing fraction of observed inputs. A) Estimated coupling coefficients plotted against true amplitude of postsynaptic currents for the grouped GLM (top, blue) and for the bilinear GLM where inputs are not grouped (bottom, red). Coupling estimates were made with different proportions of inputs (% observed) included in the model. Data from 4 cells, 5Hz conditions only. B) The correlation between the true and estimated amplitudes increases as the fraction of inputs included in the model increases for both the grouped and bilinear models. The correlation between true and estimated amplitudes for the pairwise model with the same data is shown for reference (black). Solid lines show exponential (weighted least squares) fits, and error bars denote standard error. Results are shown for 200s of data from 4 cells firing at 5Hz.

As with the pair-wise analysis above, a model that includes multiple presynaptic inputs allows accurate reconstruction of their true amplitudes. The accuracy of the reconstruction for both the grouped and bilinear models increases as more inputs are included in the model (Fig. 5). With 5% of the inputs observed, the coupling coefficients estimated by the GLM (average kernel from 0–25ms) are already well correlated with the underlying input amplitudes (R^2^ = 0.87 for the grouped model and R^2^ = 0.69 for the bilinear model). With all inputs observed this correlation increases to R^2^ = 0.94 and R^2^ = 0.82 for the grouped and bilinear models, respectively. Since the bilinear model has many more parameters, it is not unsurprising that there is more uncertainty in the parameter estimates given the same amount of data (200s in this case). However, in both the grouped and bilinear models, including multiple inputs provides more accurate reconstruction than pairwise model fit to the same data (Fig. 5B).

**Fig 6:**
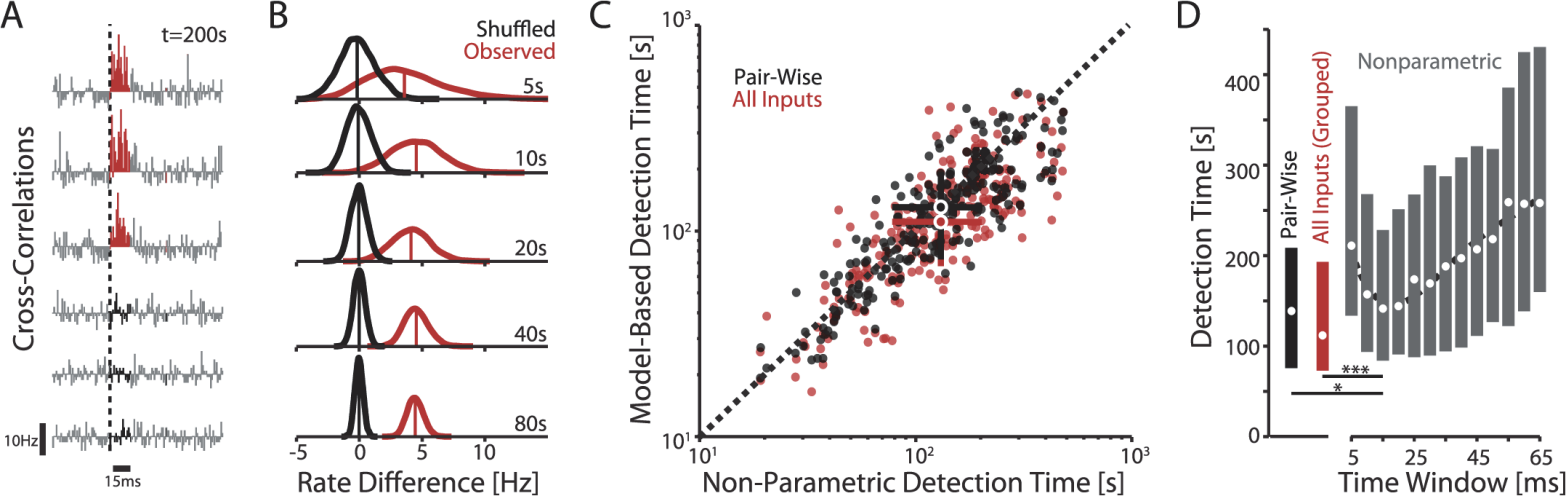
Modeling multiple inputs improves detection times. A) Schematic of a typical non-parametric test for connectivity. By bootstrapping we first generate sample cross-correlations between the spike trains of two neurons. Cross-correlograms of connected neurons show a characteristic peak (top three panels). No peak is present after shuffling the ISIs of presynaptic neuron (three lower panels). B) Distributions of spike counts in a 15 ms window following presynaptic spikes in observed (red) and shuffled (black) cross-correlograms. Vertical red and gray lines denote the distributions’ mean. These distributions can be used to test whether presynaptic spikes significantly change the probability of postsynaptic firing. A true effect will result in increasingly non-overlapping distributions as the recording time increases. C) Comparison of recording times necessary to detect connections using the non-parametric tests (data as in A and B, with 15ms window) and the model-based methods. While the model-based and the non-parametric tests perform similarly on pair-wise analyses (black dots), modeling all inputs improves the detectability of individual connections (red). Error bars denote inter-quartile range. Data from N = 4 cells. D) Detection with the non-parametric test is sensitive to the size of the time-window used for detection. However, model-based methods perform better than non-parametric test with an optimal window, 15ms in this example. Dots and error bars denote median and inter-quartile range for the strongest 25 inputs to each of 4 cells. Dashed line denotes a 4th-order polynomial fit to the medians for reference.

The increased accuracy in estimating synaptic weights using models of all inputs suggests that postsynaptic spikes might be more readily associated with or disassociated from the spiking of individual presynaptic inputs. Previous methods for detecting synaptic inputs, by using pairwise spike statistics alone, do not take advantage of this additional predictability. For instance, in the traditional, non-parametric tests the cross-correlation between spiking of two neurons is compared to the cross-correlation obtained when spike timings of the presynaptic neuron are shuffled (Fig. 6A). Using boot-strapping we can create a distribution of the total spike count in a cross-correlation over a window following presynaptic spikes (Fig. 6B). Comparison of the distributions obtained using the observed *vs* shuffled spike trains allows us to test whether an input has a statistically significant effect on the firing of the postsynaptic neuron (see Methods). As with the model-based methods, we can vary the amount of data (recording length) used for computing this statistic, and determine a minimal recording time necessary for the detection that, as expected, depends strongly on the amplitude of an input (Fig. 6C).

Using data from experiments with fully-defined input we find that, even after optimizing the detection window for the nonparametric test (Fig. 6D), the pair-wise, model-based method yields slight but significant reductions in detection time (to 94±2% on average compared to the nonparametric test with 15ms time window, paired two-sided sign test p = 0.005). A grouped model that includes all inputs yields additional reductions in detection time (to 86±4%) compared to the nonparametric approach (paired two-sided sign test p<10^−5^). Using all inputs in the grouped model also reduces detection times relative to the pair-wise, model-based tests, where spiking of only one presynaptic neuron is included in the model, while all other inputs are treated as noise (to 94±2%, paired two sided sign test p = 0.04).

One reason for the faster detection of connections between neuron pairs by model-based inference than by cross-correlation based non-parametric tests might be that the non-parametric test requires us to specify a fixed interval for counting spikes on the cross-correlogram (0–15ms is optimal here). Making this interval too long or too short will reduce the sensitivity of the test (Fig. 6D). However, the model-based methods are more robust to these differences, since the coupling kernel can flexibly account for changes in postsynaptic spike statistics produced by inputs with different time-courses. This advantage may be especially useful for reconstructing connectivity from *in vivo* data, where the shape of PSCs varies broadly across connections. Indeed, previous work has shown how model-based methods can produce a more accurate picture of connectivity compared to descriptive (cross-correlation) methods [22]. These results obtained in a fully-defined experimental setting illustrate how model-based methods can be applied to improve input detection over descriptive methods, as well.

### Prediction of spikes

**Fig 7:**
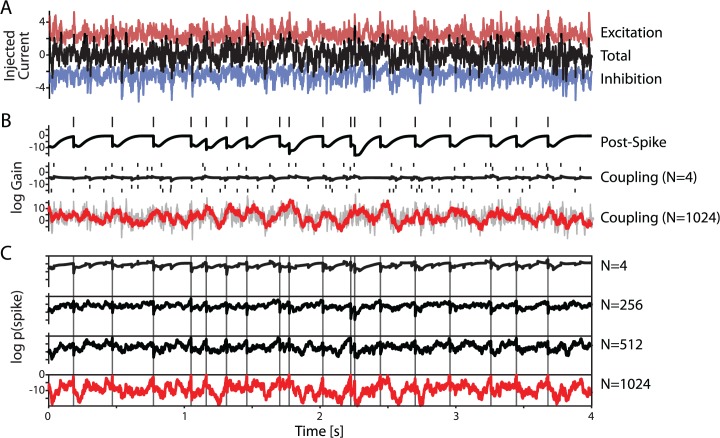
Observing more presynaptic neurons improves spike prediction. A) Injected current is a combination of simulated excitatory and inhibitory PSCs. The variance of the total current and DC component are later adjusted to induce desired amplitude of membrane potential fluctuations and firing rate. B) Postsynaptic spiking is predicted based on the post-spike history and activity of the presynaptic inputs. The post-spike history has a large effect and describes intrinsic dynamics of the spike generation mechanism. Each presynaptic input has a small effect by itself, but as more inputs are included in the model the influence of coupling increases and becomes more predictive than post-spike history. Note that, although the model does not explicitly describe voltage dynamics, the effect of coupling is correlated with the injected current (gray trace). C) As more inputs are included in the model spike prediction becomes increasingly accurate (vertical lines denote observed postsynaptic spike times).

In addition to examining the detectability of individual inputs, the fully-defined input protocol allows us to extend the basic GLM framework to predict postsynaptic spiking based on recent activity of *multiple* presynaptic inputs. The pairwise analyses above indicate that the spike times of individual presynaptic inputs are useful in predicting postsynaptic spikes. Since inputs are independent, observing more presynaptic neurons should improve the overall model accuracy. Because spike times of all N = 1024 excitatory and inhibitory presynaptic neurons composing the total input to the postsynaptic neuron (Fig. 7A) are defined, we can analyze how increasing the fraction of observed inputs improves the prediction of postsynaptic spikes.

The post-spike history term always plays a large role in predicting postsynaptic spikes (e.g. Fig. 7B, top). This term describes the strong influence that generation of an action potential has on the probability of future spikes. These dynamics are intrinsic to the spike generation mechanism and are present no matter how many inputs are included in the model.

With a small number of observed inputs the history term strongly dominates the prediction of spikes. Fig. 7B shows the time course of changes of gain that are due to the post-spike history term and due to the influence of N = 4 inputs included in the model for a typical neuron fit using the grouped model (middle plot). A linear combination of these few sparse, excitatory (spikes indicated by small bars above the trace) and inhibitory (spikes below the trace) inputs has only weak influence on the spiking, and can account for only a small portion of the variations in spike timing. However, as the number of inputs included in the model grows, their total contribution becomes comparable to that of the post-spike history term (Fig. 7B, bottom). Although the model used here does not explicitly describe the underlying fluctuations in membrane potential that result from the current injection, the contribution of the cumulative coupling terms of all inputs (N = 1024, red trace in Fig. 7B, bottom) tends to be correlated with the total injected current (Fig. 7B, bottom, gray trace, R = 0.54 for this example).

As more inputs are observed, overall spike prediction becomes more accurate. Fig. 7C shows a combined influence of the post-spike history term and an increasing number of inputs (N = 4; 256; 512; 1024) on the probability of postsynaptic spiking. With few inputs, the postsynaptic firing is dominated by post-spike effects and timing of postsynaptic spikes is predicted relatively inaccurately (AUC = 0.78 for this example with 4 inputs). With an increasing fraction of inputs included in the model, their contribution to spike prediction increases, and timing of individual spikes is predicted with progressively increasing accuracy (AUC = 0.82, 0.86, and 0.98 for 256, 512, and 1024 inputs, respectively). When all inputs are observed, the model spike probability tends to be sharply peaked around actual spike times (vertical lines in Fig. 7C), and the coupling effects contribute substantially to the rate variation (81% of the variance in the log rate is due to coupling).

**Fig 8:**
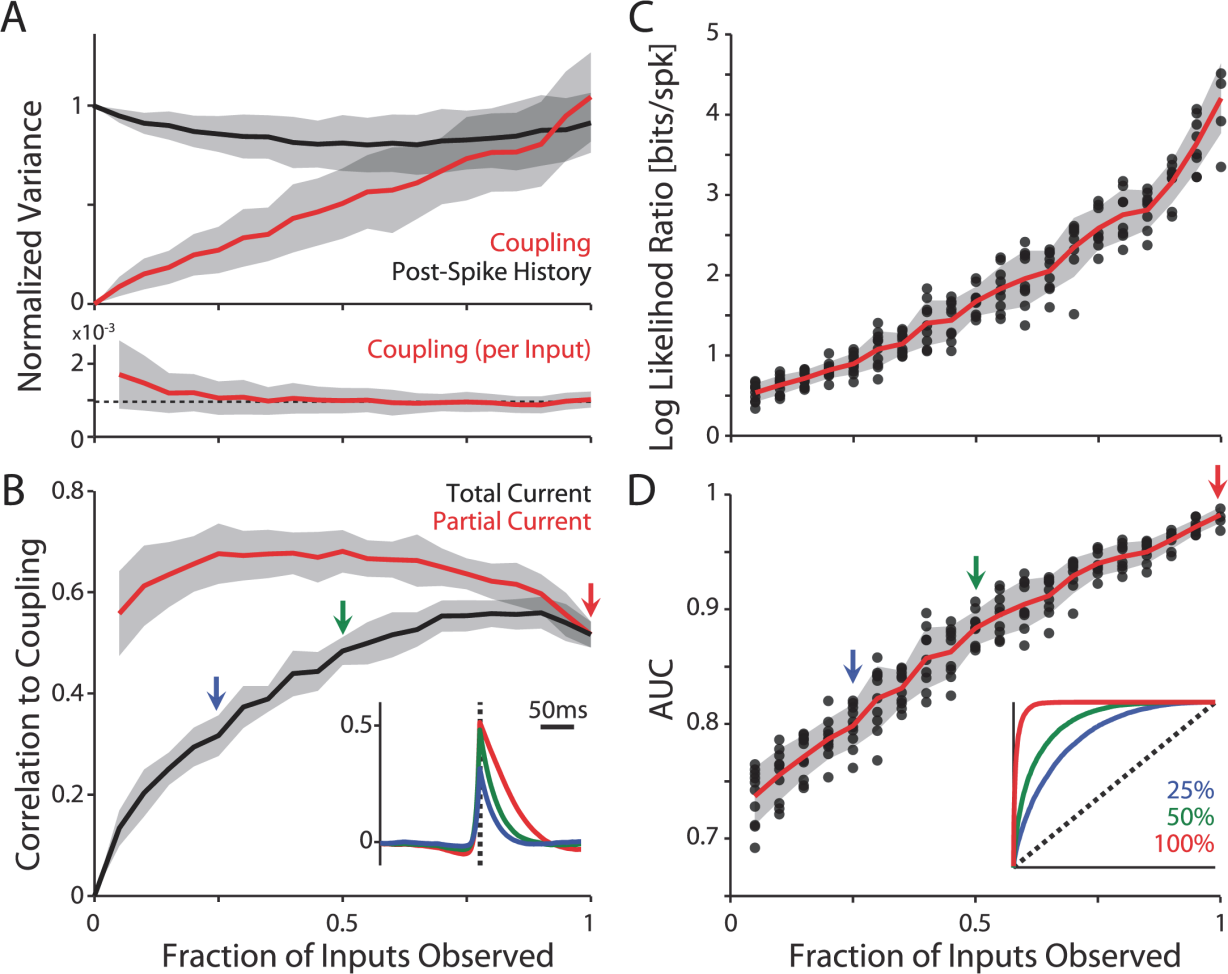
Modeling multiple inputs improves spike prediction and reveals a correlation between the model input and the injected current. A) The variance of the coupling terms and post-spike history relative to the variance of the estimated log rate, log λ(t), plotted against the fraction of inputs included in the model. As the fraction of observed inputs becomes larger, coupling describes a greater part of variations in the post-synaptic firing rate. However, the contribution of individual inputs remains relatively constant (lower) around 1/N (dashed line). B) Correlation between the coupling term and the total injected current (black), and the partial current (red, postsynaptic current that was produced by the fraction of inputs observed). Note that correlations between the coupling term and total current increase, while correlation between coupling and partial current remains consistently high. Inset illustrates the average cross-correlations between the coupling term and total current for models with 25, 50, and 100% of the inputs observed. C) Cross-validated log likelihood ratio of the full model relative to a homogeneous Poisson model as a function of the fraction of observed inputs (data for N = 4 cells, combined across postsynaptic firing rates of 1, 5, and 10 Hz). Points denote individual cells and firing rate conditions. Error bands denote standard deviation. D) Area-under-the-curve spike prediction accuracy, calculated from same data as in (C). Inset illustrates average ROC curves for model fits with 25, 50, and 100% of the inputs observed.

These qualitative results are corroborated by quantitative analysis of the contribution of the different model components to spike prediction. When only few synaptic inputs are included in the grouped model the post-spike history accounts for nearly all of the variability in the firing rate (Fig. 8A). As more inputs are modeled the contribution of coupling becomes progressively stronger, and, in the grouped model, with inclusion of ∼70–90% of the inputs it becomes as important as the intrinsic dynamics of spike generation described by the post-spike history (Fig. 8A). Note that the effect of individual inputs remains weak (Fig. 8A, bottom), but the total contribution from all inputs becomes substantial.

Similarly, as more inputs are included in the grouped model, the contribution of the coupling terms becomes increasingly correlated with the injected current (Fig. 8B, black curve). Since the total current is simply the sum of all PSCs produced by all presynaptic inputs, we can also extract the “partial” current that is the current contributed only by a fraction of the observed neurons. This partial current is fairly consistently correlated with the coupling term (Fig. 8B, red curve). The reduced correlation of both the total and the partial current with the coupling term when the fraction of observed inputs approaches 100% appears to be due to the differences in the timescales of the coupling terms under the grouped model. The coupling term tends to follow the injected current with a slightly longer timescale as more inputs are grouped together (Fig. 8B, inset).

Including larger number of inputs in the model improves spike prediction quantitatively, as well. Log likelihood ratios (relative to a homogeneous Poisson model) increase monotonically with the increasing fraction of observed inputs (Fig. 8C). Note that, unlike *in vivo* data where the LLR saturates as more neurons are observed [26], here the model accuracy shows no signs of saturation. This is likely due to the fact that the synaptic inputs simulated here are independent, while firing of presynaptic neurons *in vivo* can be much more correlated. Using ROC analysis we find that cross-validated area-under-the-curve increases from 0.72±0.01 when 5% of the inputs are observed to 0.98±0.01 in the model that includes all (100%) inputs (Fig. 8D). Assessing spike prediction accuracy with single trial data is not always intuitive. Here, an area-under-the-curve of 0.98 signifies that a randomly chosen time bin containing a spike in the recorded response will have a higher *p*(*spike*) than a randomly chosen non-spike bin 98% of the time.

**Fig 9:**
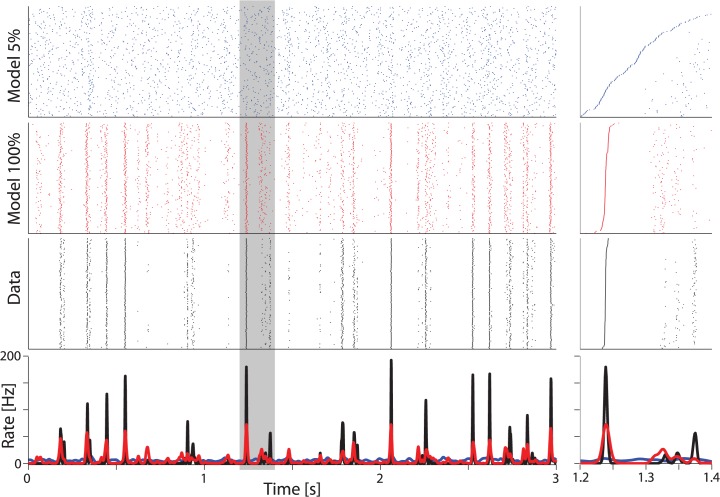
Interaction of synaptic input and intrinsic dynamics in spike prediction. Rasters show spike simulations from the model using a fixed set of presynaptic spikes as input across trials, alongside the observed spiking (“Data”) for 160 trials of a repeated stimulus segment. When only a small fraction (5%) of the presynaptic input is modeled spike predictions are highly variable from trial to trial (blue). However, when the full presynaptic input is modeled (red), the GLM more accurately reproduces the observed spike times (black). The predicted rates (bottom) match the observed PSTH, although with less precision. The panels on the right illustrate the difference in precision between the models and data for a small segment of the stimulus (gray box in left panels). Trials are sorted by time of the first spike.

To unpack the relationship between the post-spike history and presynaptic input as more inputs are included in the model we compare observed responses of a neuron to a repeated fully-defined input stimulus and responses of a grouped model to the same stimulus (Fig. 9). Here we simulate a neuron receiving the same presynaptic inputs on each trial and use the simulated post-spike history to generate model responses. Simulating multiple trials, we find that there is substantial trial-to-trial variability when only a small fraction (5%) of the inputs is used to generate the model response (Fig. 9, top). With this small number of inputs, the average response only weakly matches the peri-stimulus time histogram (PSTH) of observed spike responses (R = 0.09±0.02, PSTHs estimated by a maximum likelihood, fixed bandwidth kernel density estimator). When all inputs are modeled, trial to trial variability is substantially reduced (Fig. 9, middle), and the simulated PSTH more accurately matches the observed responses to repeated trials (R = 0.73±0.06). Similarly, the bilinear model reproduces the PSTH with R = 0.10±0.02 and R = 0.55±0.04 when 5% and 100% of the inputs are observed, respectively. Even when all inputs are modeled, the GLMs do not capture the full precision of the data (Fig. 9, right). These results suggest that, although single trial spike prediction with the GLM is quite accurate, more precise models, with additional nonlinearities [41,42] or explicit estimates of the underlying membrane potential [43], might be necessary to provide a full account of the transformations that occur as fluctuating current input leads to spiking output.

### Current-based vs conductance-based synaptic input

One potential challenge in generalizing the results of *in vitro* current-injection experiments to data collected *in vivo* is that these experiments omit several important features of real synaptic integration. *In vivo*, nonlinear dendritic integration, probabilistic release, and plasticity will all introduce certain variability in transmission at a synapse, making it more difficult to detect the connection and to estimate its strength. It is also important to note that here we use current-based synaptic input, while at real synapses currents are generated by changes of conductance. This causes the PSC amplitude to vary as a function of the membrane potential. To test whether the nature of current-based and conductance-based inputs could have a large effect on their detectability and estimation of their strength, we used post-synaptic spikes generated by two model neurons with either current-based or conductance-based synapses, and fit pairwise models to these new data.

**Fig 10:**
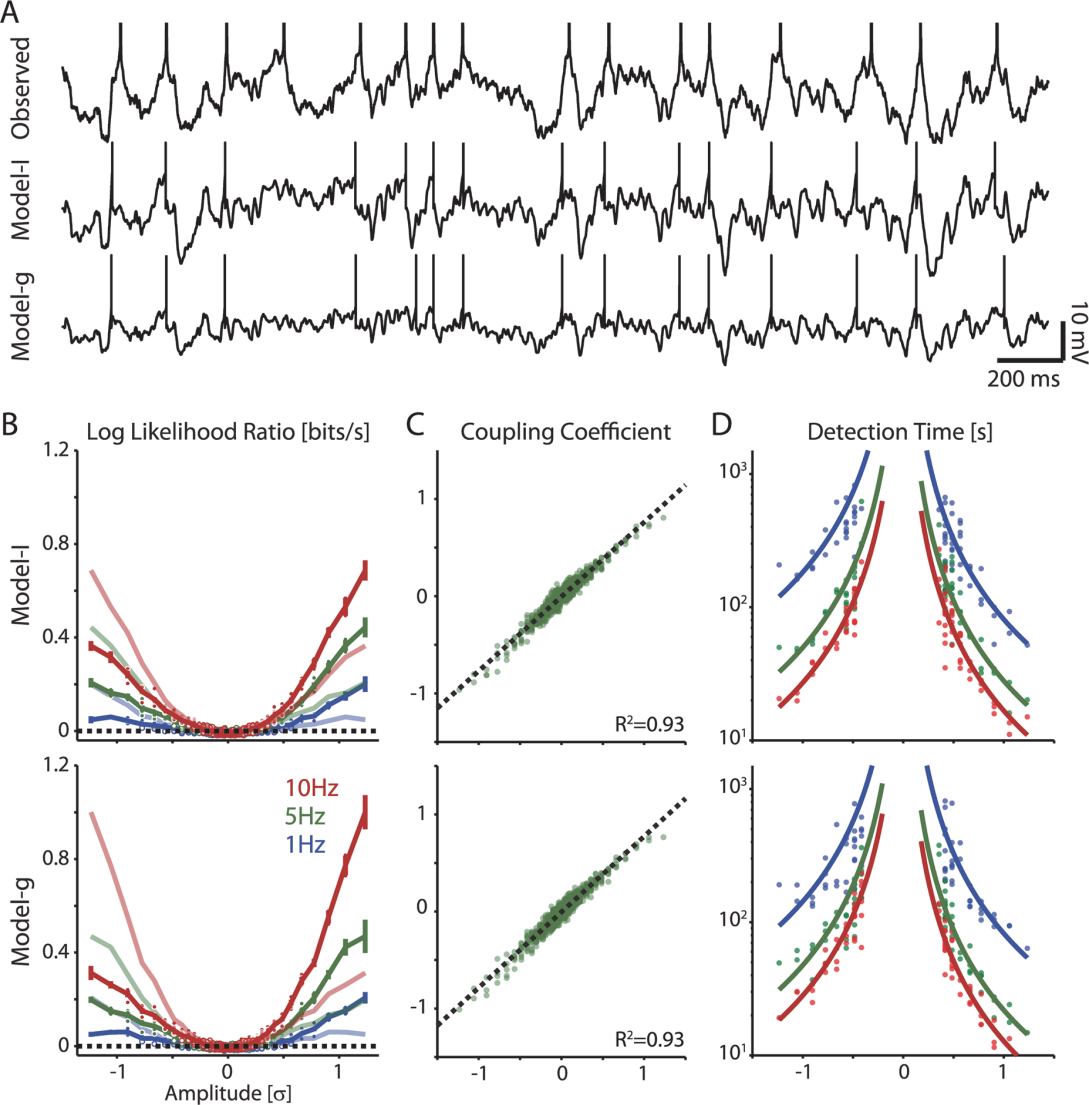
Detection of synaptic inputs in model neurons with current-based or conductance-based synaptic inputs. A) Membrane potential traces recorded in a neuron *in vitro* (“Observed”) and simulated traces for two adaptive-exponential integrate-and-fire neuron models receiving either current-based (Model-I) or conductance-based (Model-g) synaptic inputs. In all three cases, input was generated using the same set of presynaptic spike times and synaptic weights. After optimizing model parameters, both models accurately reproduce the observed voltage fluctuations and spike timing of the recorded neuron. B) Detectability of synaptic inputs of different strength in the two models. Log likelihood ratio between models with no coupling and with constant-gain coupling plotted against input amplitude, for firing rates of at ∼1Hz, ∼5Hz, and ∼10Hz, as indicated. To illustrate the more reliable detection of excitatory inputs, lighter colors show the average log-likelihood ratio curves reflected about input amplitude 0. C) Correlation between the estimated coupling coefficient and true PSC amplitudes (5 Hz data). Dashed lines denote linear fits. D) Time necessary for input detection against its amplitude, for firing rates of at ∼1Hz, ∼5Hz, and ∼10Hz. Solid curves denote fits for the function c/x^2^. Note that detectability of inputs in the models with current-based and conductance-based synapses are similar for two models and to the detectability of inputs in recorded neocortical neurons (compare to Fig. 4). Performing the same pair-wise analyses as above we find that, despite difference in the form of the input, once spike statistics are matched, the detectability of inputs of different strengths (A), estimation accuracy for the 5Hz condition (B), and detection times (C) are comparable to the experimental data. As before filled dots in (B) denote cases where Model 2 with coupling, provided significantly better fits than Model 1 with no coupling (Chi-square test, p<0.05). Error bars denote SEM. Dashed lines in (C) denote linear fits. Solid curves in (D) denote fits for the function c/x^2^.

To simulate postsynaptic neurons that match the Layer 2/3 cells recorded *in vitro*, we fit two adaptive exponential integrate-and-fire models: one with current-based synapses and one with conductance-based synapses [44]. Using the same presynaptic spike times and weights delivered to the observed neurons, we then optimize the parameters of these models to match both the observed membrane potential and spike timing (Fig. 10A, see Methods). After optimization, the sub-threshold fluctuations of the models reproduce the observed fluctuations to 4.1mV RMSE – with similar accuracy between current-based and conductance-based models. Overall spike statistics (post-synaptic inter-spike interval distributions and cross-correlations) were well-matched between the models and data (S1 Fig.), and the spike coincidence factor [45] with Δ = 4*ms* averaged Γ = 0.35 for the current-based models and with the conductance-based models performing slightly worse at Γ = 0.31 (S2 Fig.).

Using the simulated spike trains from the two adaptive exponential integrate-and-fire model neurons, we performed the same pair-wise analyses for estimating synaptic amplitude and detection time that we used for the real data (Fig. 10B-D). As with the experimental data, we find that, in both the current-based and conductance-based models, higher amplitude synapses are easier to detect and there is an asymmetry between excitatory and inhibitory inputs with inhibitory inputs being more difficult to detect than excitatory inputs of the same magnitude. As before, the postsynaptic firing rate has a large effect on detectability and estimation accuracy with higher rates resulting in faster detection of inputs. Estimation accuracy for the current-based model, in this case, was R^2^ = 0.85 for 1Hz output, 0.93 for 5Hz output, and 0.96 for 10Hz output (for clarity only 5Hz condition is shown in Fig. 10C). The conductance-based model yields similar results with R^2^ = 0.84, 0.93, and 0.96 for the three output rates. Finally, we find that detection times decrease as ∼c/x^2^ with increasing input amplitudes, and are shorter for higher post-synaptic firing rates. Together, these results show that the form of synaptic input – conductances *vs* currents – does not substantially alter our results once spike statistics are matched.

## Discussion

Here we use *in vitro* current injection experiments to assess how well functional connectivity inferred from spikes corresponds to underlying synaptic inputs and to examine the limits of postsynaptic spike prediction from presynaptic spike times. Our results show that 1) Simulated synaptic inputs with amplitudes >0.25σ_noise_ can be reliably inferred from several minutes of spiking activity of pairs of neurons. Stronger connections can be detected even faster, but the amount of data required for detecting weaker connections increases rapidly. Thus, in typical experiments only a subset of connections can be detected, with a low amplitude limit depending on recording time and firing rate. 2) The inferred coupling strength is strongly correlated with the true amplitudes of synaptic inputs. 3) Excitatory inputs are more readily detected than inhibitory. 4) Detectability of changes of synaptic weights follows same rules and has same limitations as detection of individual synaptic connections. 5) Observing and modeling larger fractions of the presynaptic input improves model accuracy and reduces the necessary recording time for detecting inputs. Thus, model-based inference of functional connectivity allows accurate detection of individual inputs, reconstruction of synaptic amplitudes, and prediction of postsynaptic spiking.

Experiments with injection of currents composed of mixtures of input signals provide a flexible experimental framework for exploring how synaptic input is translated into output postsynaptic spiking. These recordings provide a link between properties of synaptic connections typically measured in intracellular experiments *in vitro*, such as PSC amplitude, and estimates of connectivity made from *in vivo* extracellular recordings, such as inferred functional connectivity and coupling strength. We have used a real spike generation mechanism of an actual neuron to study spike responses to artificial PSCs with realistic time courses, distribution of weights and firing statistics of presynaptic neurons [46,47], consistent with experimental observations. Injected currents induced membrane potential fluctuations typical for *in vivo* activity [8,48,49]. However, we have also made some simplifying assumptions and neglected to model many known effects, such as nonlinear dendritic integration [50], short-term plasticity [51], possible input correlations [52], probabilistic release, channel noise [53], and the fact that actual synapses are conductance-based [54,55]. Since these effects can have a substantial impact on spike statistics, future experimental work and statistical modeling may yield improvements over the current methods. These additional experimental parameters will dramatically increase the complexity of future experimental results and their interpretation. Here we have neglected these effects in order to simplify our analyses and determine the basic constraints of what can be inferred about simulated synaptic connectivity from spiking of neuronal ensembles. Although these experimental simplifications may limit the extent to which our results can be directly generalized to extracellular recordings, the trends described here provide concrete links between synaptic currents and the detectability of synaptic inputs. Applying model-based methods to spike trains generated by model neurons fit to our observed data, we found that our results are robust to both the form of input (conductance-based *vs* current-based synapses) and the spike generation mechanism (integrate-and-fire *vs* biophysical in real neurons).

In addition to these physiological caveats, it is important to note that there is also room for improvement in modeling. The model-based methods used here are based on a tractable class of rate models and fail to describe some fundamental aspects of spike statistics. Here we used a common GLM approach where neurons are assumed to emit spikes according to a Poisson random variable with a rate that is determined by recent pre- and postsynaptic spiking. While these models can describe the auto- and cross-correlations present in the data, neurons often have more complex nonlinear behavior [56,57] and generate spikes much more reliably than Poisson processes [58]. In general, rate models may fail to capture some of the important nonlinear dynamics of real neural systems [59]. However, several statistical models have been developed that explicitly aim to describe the underlying membrane potential dynamics [60,61] and tend to yield more accurate spike prediction. Although inference from spike trains is more difficult in these models, they are able to model the high reliability of spikes in many experimental settings [43] and provide detailed insight into the membrane dynamics and changing conductances underlying the observed spiking [62]. Including coupling between neurons in these voltage-based models, as latent synaptic conductances, may lead to improved detection, estimation, and tracking of inputs from spikes.

Despite these caveats, GLM-based estimation of functional connectivity offers some important advantages over previous methods. Descriptive statistics of functional connectivity [18,19,63,64] and recent extensions [65] are widely applied tools for understanding interactions between neurons. However, as many previous studies have noted, correlations that are not due to direct monosynaptic connections can confound estimates of connectivity and compromise the interpretation of cross-correlograms [20,29]. Common-input, the dynamic effects of post-spike history on future spikes, poly-synaptic effects, and changing synaptic weights will all affect basic pair-wise spike statistics. One key advantage of statistical inference model-based methods, over descriptive techniques, is that the many factors contributing to postsynaptic spiking can be modeled simultaneously and, to some extent, disentangled.

Our initial results show that estimates of synaptic coupling by statistical-inference model are sensitive to changes in synaptic strength, and thus can provide a tool for assessing plasticity of synaptic connections from neuronal spiking. We demonstrated that detecting a synaptic weight change that might occur after a specific experimental intervention, such as tetanization, requires only moderately more data than detecting the presence of a connection. However, statistical methods that can infer synaptic changes from extracellularly recorded spike trains could also be applied to situations where change-points are unknown or not defined, for instance, when plasticity is a result of ongoing natural activity. New statistical methods are beginning to address these issues by explicitly modeling ongoing plasticity [66,67], considering state-changes with unknown change points [68], and providing more flexible descriptions of time-varying neural dynamics [69–72].

In our analysis of stationary coupling, the use of a broad range of synaptic amplitudes composing the injected currents allowed us to test the limits of detectability and determine how much data is necessary to detect the effect of presynaptic spiking on postsynaptic spike statistics. We find that detection time for an input of amplitude *x* is approximately proportional to 1 / *x* ^2^ and also depends on firing rates. Importantly, inhibitory inputs are more difficult to detect than excitatory inputs of the same amplitude, and inhibition required ∼30% more data for detection with pair-wise tests. We further show that when an increasing fraction of presynaptic neurons can be included in the model, spike prediction accuracy increases and less data is necessary for detection of individual connections. Here we found that with ∼15min of data and the pre- and postsynaptic cells firing at ∼5Hz, synapses could only be reliably detected when they had amplitudes > 0.25σ. This corresponds to PSCs amplitudes of 18–27 pA, which is in the range of average EPSCs amplitudes in L2/3 cells (15–25 pA, [73]). These results suggest that, to fully map functional connectivity estimated from spikes to actual physiological connectivity, longer recording lengths may be necessary to detect weak connections. At the same time, pairs of neurons with strong synapses or high firing rates may be evident even in short *in vivo* recordings.

Previous work has shown that modeling partial, putative presynaptic input can improve both spike prediction and decoding [26,30,74]. Here we consider a limiting case where the input is fully controlled during the experiment. We find that a basic rate model (GLM) can provide surprisingly accurate spike predictions and approximately capture the true input current. These findings suggest that models of functional connectivity may begin to provide insight into actual synaptic effects as modern techniques allow recording of simultaneous spiking from increasing numbers of neurons *in vivo*.

*In vitro* current injection has been used to study temporal integration of presynaptic spikes [3,37], the effects of input synchrony on spiking [75], and signal propagation through networks [76]. Here we focused on the detectability and identification of individual inputs from simultaneously recorded spike trains. Our results highlight some of the important scientific possibilities offered by the use of statistical methods for understanding large-scale interactions between neurons. Modeling the interplay between post-spike history effects and presynaptic input will be essential to describe biophysical diversity within [77,78] and across [79,80] cell types. Extending both the statistical and experimental tools used here will provide a deeper understanding of the transformations of synaptic inputs into a neuron’s spiking output.

## Methods

### Ethics statement

All animal use procedures were approved by the institutional animal care and use committee at the University of Connecticut, and conform to the principles outlined in the Guide for the Care and Use of Laboratory Animals (National Institutes of Health publication no. 86-23, revised 1985).

### Electrophysiology and current injection

*In vitro* intracellular recordings were made from layer 2/3 pyramidal neurons in slices of rat visual cortex (Wistar rats, P17-P30), as described in previous work [3,39]. The solution used during the preparation of the slices and during the recordings contained (in mM) 125 NaCl, 2.5 KCl, 2 CaCl_2_, 1 MgCl_2_, 1.25 NaH_2_PO_4_, 25 NaHCO_3_, 25 D-glucose and was bubbled with 95% O_2_ and 5% CO_2_. Whole-cell recordings were made with patch electrodes (4−6 *M*Ω) filled with K-gluconate based solution (in mM: 130 K-Gluconate, 20 KCl, 4 Mg-ATP, 0.3 Na_2_-GTP, 10 Na-Phosphocreatine, 10 HEPES) using the bridge mode of a Dagan BVC-700A amplifier (Dagan Corporation, USA). Data were digitized at 20 kHz (Digidata 1440A, Molecular Devices, USA) and stored for further processing.

To systematically study the detection and tracking of synaptic weights we injected neurons with partially-defined or fully-defined synthetized current. 1) Partially-defined current was produced by a single simulated, excitatory presynaptic input immersed in fluctuating noise. Several amplitudes of artificial EPSCs were used in these experiments. 2) Fully-defined current was produced by the firing of a large population of simulated presynaptic excitatory and inhibitory neurons whose postsynaptic currents (PSCs) sum to mimic fluctuating, naturalistic input.

### Experiment 1. Partially-defined input: Artificial EPSCs immersed in fluctuating noise

Current for injection in this first set of experiments was composed of 1) a fluctuating component *ση*(*t*), where *η*(*t*) is a standardized (zero mean, unit variance) Ornstein-Uhlenbeck (OU) process with a correlation time of *τ* = 5*ms* rescaled to have standard deviation σ, 2) artificial EPSCs of several different amplitudes: 0.1, 0.2, 0.25, 0.3, 0.5, 1.0 and 1.5 of the noise standard deviation σ, and 3) a DC component tuned to maintain a desired firing rate, around ∼5 Hz. Seven cells were injected with (0.5, 1.0, 1.5 σ inputs), one cell with (0.2, 0.3, 0.5, and 1 σ inputs), and two cells with (0.1, 0.25, 0.5, 1.0 σ inputs). This approach was similar to that used in previous work [2,3]. Note that this approach based on injection of current through the intracellular electrode ignores the transformation of synaptic conductances into postsynaptic currents, as well as effects of dendritic integration of synaptic inputs. However, the injected fluctuating current reproduces well the membrane potential fluctuations recorded in the soma of neocortical neurons *in vivo* [8,48,49]. Presynaptic spike timing for the artificial EPSCs was generated by a gamma renewal process (shape *k* = 2, scale *θ* = 2.5). This corresponds to a neuron firing at a rate of 5Hz with more regularity (*CV* ≈ 0.7) than a Poisson process. Spikes were then convolved with a synaptic integration kernel generated by a difference of exponentials – rise time of 1ms and decay time of 10ms – to generate an EPSC trace. This kernel was previously used in [3]. *In vivo*, there is substantial variation in spike statistics [80,81] and dendritic integration [82,83]. Although a complete exploration of the parameter space is beyond the scope of these experiments, the values used here were chosen to be consistent with *in vivo* and *in vitro* observations.

Current was injected in episodes of 46s, with intervals of 40–90s between the episodes. We recorded from 10 cells with 10–60 episodes recorded from each cell.

The analysis (described below) assumes that the responses are stationary, such that the files can be reordered and concatenated without loss of generality. That is, if episodes are recorded with aEPSC amplitudes 0.1, 0.2, 0.5, 0.2 σ, we assume that we can examine an amplitude change of 0.4 σ by concatenating files 1 and 3 and that concatenating files 2 and 4 is equivalent to a single longer recording with 0.2 σ input. In all cases, the intervals between episodes, where there was no postsynaptic spiking, as well as the first and last 3s of each record were excluded from the analysis. In this first set of experiments we do not consider the effects of IPSCs.

### Experiment 2. Fully-defined input produced by a population of spiking neurons

In a second set of experiments, rather than injecting current composed of a single input plus noise, we aim to model complete presynaptic input by simulating the spiking of large number of presynaptic neurons. Several factors determine the statistics of the injected current: 1) the statistics of presynaptic spiking, 2) the amplitudes of synaptic “weights”, and 3) the time-course of the PSCs. Here we make several simplifying assumptions. We assume that population of presynaptic neurons consists of an identical pool of gamma renewal processes, 50% of which are excitatory and 50% of which are inhibitory, and we assume a log-normal distribution of synaptic weights based on observations from paired *in vitro* cortical recordings [46]. Finally, we generated the PSCs as a difference of exponentials with a rise time of 0.5ms and decay time of 5ms.

Thus, the model parameters are *N* (the number of presynaptic neurons), *k* and *θ* (the shape and scale parameters for the homogeneous Gamma renewal processes), *μ* and σ (the shape and log-scale parameters of the log-normal amplitude distribution), and *τ*_1_ and *τ*_2_ (the time constants of the artificial PSCs). Here we use *N* = 1024 with amplitudes drawn pseudo-randomly from a discrete approximation to the log-normal distribution described in [46] (*μ* = 0.702, σ = 0.9355). Absolute PSC amplitudes are constrained to 32 discrete, log-spaced values to simplify the analysis, and the proportion of amplitudes in each bin is exact to maintain excitatory-inhibitory balance. Excitatory and inhibitory inputs are assumed to have the same distribution, differing only by the sign. We use *k* = 2, *θ* = 2.5 for the gamma renewal processes and resample short (<10ms) ISIs to avoid strongly overlapping PSCs.

It is important to note that these artificial PSCs only partially capture the dynamics of real synaptic currents, which exhibit synaptic noise, as well as, short-term depression and facilitation. Moreover, this basic population spiking model does not capture all of the details of cortical circuitry. There are known differences in the spike statistics and time course of PSCs for different cortical cell types [80]. However, this model maintains balanced, approximately OU statistics and allows us to examine the detectability of a broad range of synaptic weights. The simulation parameters here were chosen with the goal of making inputs physiologically realistic, but structured enough to analyze statistically.

Using these parameter settings we recorded an additional 5 cells where the DC component was tuned to maintain a desired firing rate. Three cells were recorded around ∼5 Hz, while two cells were driven at multiple firing rates: ∼1 Hz, ∼5 Hz and ∼10 Hz.

### Inferring functional connectivity from spikes

For detection of synaptic inputs from spiking, we use a basic statistical model that describes the probability of postsynaptic spikes as a function of the presynaptic spike timing – a generalized linear model (GLM) with Poisson observations. For a single presynaptic input we assume

$$\lambda \left(t\right)=\exp\left(b_{0}+\sum_{k=1}^{K}b_{post,k}f_{k}\left(n_{post}(t)\right)+\sum_{k=1}^{K}b_{pre,k}f_{k}\left(n_{pre}(t)\right)\right)$$ (1)

$$n_{post}(t)∼Poisson\left(\lambda (t)\right).$$

We aim to predict postsynaptic spiking *n*_*post*_(*t*), assuming that spikes are generated as Poisson random variables with a rate *λ*(*t*). This rate is determined by a linear combination of a baseline firing rate *b*_0_, the influence of spiking history, that is the previous spikes of the postsynaptic neuron, parameterized by *b*_*post*_, and a term that describes the effect of the input from presynaptic spikes, parameterized by *b*_*pre*_. A set of basis functions *f*_*k*_ provides a smooth expansion of each of these effects. The parameters of this model can then be readily estimated by maximizing the log-likelihood [84]

$${\hat{b}}_{MLE}={\mathrm{arg max}}_{b}\sum_{t}\left(n(t)\log\lambda (t)-\lambda (t)\right).$$ (2)

In the results presented here we often summarize the effect of coupling with a single “coupling weight”, defined as the average kernel over the first 25ms: ${\langle \sum_{k=1}^{K}b_{k}f_{k}\left(\tau \right)\rangle }_{0<\tau <25}.$.

This basic GLM can then be extended to include multiple inputs by using

$$\lambda \left(t\right)=\exp\left(b_{0}+\sum_{k=1}^{K}b_{post,k}f_{k}\left(n_{post}(t)\right)+\sum_{i=1}^{N}\sum_{k=1}^{K}b_{i,k}f_{k}\left(n_{i}(t)\right)\right).$$ (3)

In this case, with *O*(*kN*) parameters, rather than straight-forward maximum likelihood estimation, regularization is important to prevent over-fitting. Here we use L1-regularization and find the maximum a posteriori (MAP) parameters estimates

$${\hat{b}}_{MAP}={\mathrm{arg max}}_{b}\sum_{t}\left(n(t)\log\lambda (t)-\lambda (t)\right)-\eta \sum_{i=1}^{N}\sum_{k=1}^{K}\left|b_{i,k}\right|.$$ (4)

The regularization hyper-parameter is optimized by maximizing cross-validated log-likelihood, and no regularization is performed on the baseline or post-spike history terms. Here we use 10-fold cross-validation both to optimize the hyper-parameter and evaluate model accuracy (see below).

With L1-regularization we assume that input weights are exponentially distributed *a priori*, and this assumption is useful in that it prevents overfitting and leads to sparse solutions where many input weights *b*_*i*,*k*_ are set to 0. This assumption is useful for analysis of *in vivo* data, since only a small number of potential presynaptic inputs are likely to actually be connected to the postsynaptic neuron, so such sparse solutions are desirable. Other regularizers, such as spike-slab, group-L1, or even the log-normal prior used to generate the input weights here, may produce more accurate estimates. However, the fact that the log-posterior is concave with L1-regularization provides the additional advantage of a unique, global optimum. For both MLE and MAP (L1-regularized) optimization we use scaled sub-gradient maximization using the L1General toolbox [85].

Even with regularization, fitting a model with 1024 inputs is computationally prohibitive. Here we use two alternative simplifications: 1) a grouped GLM where inputs of the same weight are grouped into a single covariate, and 2) a bilinear extension of the GLM where the inputs are constrained to take the same shape.

The grouped model takes advantage of the fact that the simulated amplitudes are highly structured. Namely, groups of presynaptic neurons have identical amplitudes distributed according to a discrete approximation of the log-normal distribution [46]. We can thus collect inputs into groups to simplify the analysis from Eq. (3) – instead of *N* = 1024 simulated presynaptic input neurons, we can study an equivalent system with only *N* = *G* inputs, where *G* is the number of amplitude “groups” (G = 64 used in our simulations) by combining spike trains

$$n_{g}\left(t\right)=\sum_{i\in g}n_{i}(t).$$

Fitting a GLM for this reduced set of inputs as

$$\lambda \left(t\right)=\exp\left(b_{0}+\sum_{k=1}^{K}b_{post,k}f_{k}\left(n_{post}(t)\right)+\sum_{g=1}^{G}\sum_{k=1}^{K}b_{g,k}f_{k}\left(n_{g}(t)\right)\right)$$

is much more computationally tractable than modeling all individual inputs when *N* ≫ *G*. Here we make use of this group simplification to examine how detectability and overall model accuracy are affected by the fraction of observed inputs. In this case we randomly sample a fraction *p* of the total inputs *N* and include these “observed” presynaptic spikes in a grouped-GLM as described above.

Note that, in most experimental settings, such group membership would be unknown, and a large number of unconnected pairs would be likely present. The results from the grouped model likely underestimate parameter uncertainty and overestimate spike prediction accuracy compared to the full estimation problem. However, accurately estimating connectivity using models with O(N) parameters is not a trivial problem for N>1000; structured regularization techniques [86] and detailed model comparison [34,87] will be essential as the number of connections in these types of models increases.

Here as a point of comparison for the grouped model we also use a bilinear model that models individual inputs with separate parameters, but restricts all inputs to have the same shape. Here, rather than *K* parameters per input, there is only a single “weight” parameter *w*_*i*_ per input.

$$\begin{array}{l} \lambda (t)=\exp\left(b_{0}+\sum_{k=1}^{K}b_{post,k}f_{k}\left(n_{post}(t)\right)+\sum_{i=1}^{N}w_{i}f\left(n_{i}(t)\right)\right)\\ f(\cdot )=\sum_{k=1}^{K}b_{k}f_{k}(\cdot ) \end{array}$$

In this case we fit the model by coordinate ascent – alternating between maximizing the posterior with respect to the input weights while holding the shape constant and maximizing the likelihood (no regularization) with respect to the shape while holding the weights constant. This approach tends to work well in practice [66,88], and effectively approximates the full model, which would have O(Nk) parameters, with a model with O(N+k) parameters.

For all models we assume the basis {*f*_1_…*f*_*K*_} to be a set of gamma distributions *t* ^*n*^ exp(−*t* / *m*) / Z where *n* = {0…*K* −1}^*s*^ and *Z* denotes normalization by the maximum. For the post-spike history term and coupling terms during pair-wise analyses we use *m* = 25*ms*, *s* = 1.2, and *K* = 8. In the full model, this same *K* = 8 basis is used for the post-spike history, but for simplicity coupling terms use a reduced basis with *m* = 45*ms*, *s* = 1.5, and *K* = 4. These basis sets allow us to fit a variety of band-limited, smooth functions on a range ∼0–500ms with faster variation near 0ms. Similar bases have been used in previous work [30,86]. Together with the baseline parameter, these settings give 9 total parameters for Model 1, 17 for Model 2, 25 for Model 3, 265 for the full grouped model with all inputs, and 1037 parameters for the full bilinear model with all inputs.

### Quantifying accuracy and detecting functional connections

Given this model framework, we can now examine whether the effect of presynaptic spiking on postsynaptic spiking is detectable. In general, if we have two models *H*_1_ and *H*_2_ with Poisson observations the log likelihood ratio is given by

$$LLR\left(H_{1},H_{2}\right)=\left[\sum_{t}n(t)\log\lambda_{1}(t)-\lambda_{1}(t)\right]-\left[\sum_{t}n(t)\log\lambda_{2}(t)-\lambda_{2}(t)\right]$$ (5)

where the two models have conditional intensities defined by *λ*_1_ and *λ*_2_ (log base 2 is used *LLR*(*H*_1_,*H*_2_) * log2 when reporting bits). Importantly, the log likelihood ratio quantifies the relative accuracy of the two models. For instance, when *H*_2_ is a homogeneous Poisson model that only describes the mean firing rate, the log likelihood ratio quantifies how much more accurately spikes are predicted by the model *H*_1_ over just predicting the mean.

An important concept in functional connectivity analysis is whether or not an input is “detectable.” One approach is to define an input as detectable if the effect *b*_*pre*_, from the model in Eq.1, results in a cross-validated log likelihood ratio >0 when compared to the nested model with *b*_*pre*_ = 0. A second approach is to use an explicit likelihood-ratio test (without cross-validation). This test makes use of the fact that, for nested models, log likelihood ratios are approximately *χ* ^2^ distributed with *df*_2_ – *df*_1_ degrees of freedom when *df*_1_ and *df*_2_ denote the number of free parameters in *H*_1_ and *H*_2_, respectively. We then consider the effect of the presynaptic input to be statistically significant when the *χ* ^2^-test gives *p* < 0.05. Here we base “detection time” on the minimum amount of recorded data (averaged over blocks length *T*) needed to satisfy the cross-validated LLR criterion.

In deciding whether a connection is present or not, it may be useful to compare the assumptions of the cross-validated log likelihood ratio and the un-cross-validated, explicit likelihood-ratio test. Especially for models with many parameters (i.e. the bilinear model with many presynaptic inputs), cross-validation is essential to avoid over-fitting. Even though estimating confidence intervals for test-set log-likelihood is problematic [89], defining “detectability” qualitatively, based on generalization performance, is more appealing in larger models. Here we use a convention of reporting and plotting the cross-validated LLR even for simple, pairwise models. In evaluating statistical significance, we limit our analysis to the pairwise models and use the un-cross-validated likelihood-ratio test.

The likelihood ratio provides one, perhaps unintuitive, notion of accuracy. Although the models used here assume Poisson observations, potentially allowing >1 spike per bin, we can also perform ROC (receiver operating characteristic) analysis by limiting our predictions to no-spike *p*(*n*(*t*) = 0|*λ*(*t*)) *vs* spike (1 – *p*(*n*(*t*) = 0|*λ*(*t*))) classification. A bin size of 1ms is used for all analysis, such that, in practice, more than one postsynaptic spike per bin never occurred.

### Detection of weight changes

The model, tests for detectability, and accuracy analyses described above are generic tools for model-based estimation of spike train statistics and have been applied to a variety of *in vivo* extracellular data [22], in which the underlying synaptic weights are unknown. The data collected here, with the controlled synaptic input, offer several unique opportunities to study how functional connectivity estimation is related to synaptic input.

In addition to comparing the estimated model parameters to the known PSC amplitudes and comparing components of the model to the injected current, we can also examine the detection of changes of synaptic inputs. We assume that responses to injected current containing PSCs of different amplitudes can be concatenated in order to study putative changes in synaptic strength. As with the detection of connections, here we define a weight *change* as detectable if modeling the effects *b*_*pre*,*t*<*t*′_ and *b*_*pre*,*t>t*′_ around a known change-point *t*′ results in a cross-validated log likelihood ratio >0 when compared to a model with a single coupling effect *b*_*pre*_ for all observations.

### Non-parametric testing on functional connections

The model-based tools described so far provide a flexible framework for studying detectability of functional connections. However, one traditional method for determining whether a functional connection is present is to examine the cross-correlations between two spike trains. Using boot-strapping we can generate a distribution that reflects the expected rate change in the postsynaptic firing following a presynaptic spike, as well as the uncertainty in those rate changes. We then construct a null distribution by measuring the cross-correlations in data where the presynaptic ISIs have been shuffled. By taking differences between samples of the observed and shuffled distributions we can then estimate a p-value: the probability of observing, by chance, a rate change at least as extreme as the one actually observed.

To compare this nonparametric test with the model-based framework, we define a test statistic based on the average cross-correlation in a range of lags 0 ≤ *τ* ≤ *τ*′ ms following the presynaptic spikes

$$r=\frac{1}{\tau '}\sum_{\tau =1}^{\tau '}\frac{1}{T-\left|\tau \right|}\sum_{t}n_{pre}(t)n_{post}(t+\tau )$$

After computing this statistic for bootstrap samples of the observed data *r*_*obs*_ and data with shuffled ISIs *r*_*shuff*_, we can test against the null-hypothesis that there was no difference between observed and shuffled statistics. The null hypothesis is rejected at a confidence level *α*, when *P*(*r*_*obs*_ – *r*_*shuff*_ > 0) > 1 – *α* / 2 or 2 *P*(*r*_*obs*_ – *r*_*shuff*_ > 0) < *α* / 2.

Here we use *τ*′ = 15ms with 1ms bins and *α* = 0.05. To compute detection times we use 4096 total samples for each distribution with recording length *T*, aggregated over 16 randomly selected time blocks.

### Comparing current and conductance-based inputs with simulated neurons

Our *in vitro* data were collected by injecting current into L2/3 pyramidal cells, where the current is generated by a sum of *fixed* post-synaptic currents from simulated pre-synaptic neurons. Since real synaptic currents are generated by changing conductances, it is possible that our detection results overestimated the detectability and estimation accuracy of synaptic weights from spikes.

To examine this potential confound we simulate an adaptive exponential integrate-and-fire model neuron in two situations: receiving either current-based or conductance-based synaptic inputs. The adaptive integrate-and-fire model takes the general form

$$\begin{array}{l} C\frac{dV}{dt}=-g_{L}(V-E_{L})+g_{L}\Delta_{T}\exp\left(\frac{V-V_{T}}{\Delta_{T}}\right)-w+I_{0}+I_{syn}(t)\\ \tau_{w}\frac{dw}{dt}=a(V-E_{L})-w \end{array}$$

Where the dynamics of the membrane potential *V* depend on the capacitance *C*, leak conductance *g*_*L*_, resting potential *E*_*L*_, an adaptation variable *w*, DC current input *I*_0_, and fluctuating synaptic currents *I*_*syn*_(*t*). The behavior of the membrane near the spike threshold *V*_*T*_ is determined by the exponential nonlinearity$g_{L}\Delta_{T}\exp\left(\frac{V-V_{T}}{\Delta_{T}}\right)$, and the adaptation variable has its own dynamics determined by *τ*_*w*_ and *a*. As with other integrate-and-fire models, spikes occur when the membrane potential crosses a threshold *V*_*T*_, after which the potential is immediately reset to *V*_*reset*_. After spiking the adaptation term is also updated instantaneously with *w* ← *w* + *b*. Here the exponential nonlinearity and adaptation term allow us to better describe the post-spike and near-threshold behavior of the observed neurons.

To examine the effects of current-based and conductance-based inputs we use the same set of simulated pre-synaptic spikes as were delivered to the observed neurons. For current-based inputs we know *I*_*syn*_(*t*) exactly. Given the simulated presynaptic spike times *n*_*i*_(*t*), PSC kernel *p*, and weights for each synapse *v*_*i*_ these currents were generated as

$$I_{syn}(t)=\sum_{i}v_{i}\sum_{\tau }\left(n_{i}(t)*p(t-\tau )\right).$$

To modify the synaptic inputs for changing conductances we split the inputs into excitatory and inhibitory contributions with two separate reversal potentials

$$I_{syn}(t)=-g_{E}(t)(V(t)-E_{E})-g_{I}(t)(V(t)-E_{I}).$$

The conductances are then given by $g_{E}(t)=a_{E}\sum_{i\in E}v_{i}\sum_{\tau }\left(n_{i}(t)*p(t-\tau )\right)$ and $g_{I}(t)=a_{I}\sum_{i\in I}v_{i}\sum_{\tau }\left(n_{i}(t)*p(t-\tau )\right)$ where two additional parameters *a*_*E*_ and *a*_*I*_ weight the excitatory and inhibitory contributions.

Here we choose standard values for *E*_*L*_ = −70*mV*, *E*_*E*_ = 0*mV*, and *E*_*I*_ = −90*mV* and optimize all other parameters to match the membrane potential fluctuations and spike timing of the observed neurons. For the current-based models this formulation leaves the parameters {*C*,*g*_*L*_,*V*_*T*_,*V*_*reset*_,Δ_*T*_,*τ*_*w*_,*a*,*b*} to be optimized. For the conductance-based models, since we want the average PSC for each presynaptic input to match the original inputs, we introduce an additional constraint that the excitatory and inhibitory input be balanced: *a*_*I*_ < *V*(*t*) – *V*_*I*_ > = – < *V*(*t*) – *V*_*E*_ *> a*_*E*_. Since *I*_*syn*_(*t*) has 0 mean, we approximate the expectations using the equilibrium voltage far from threshold: *V*_*eq*_ = *V*_*L*_ + *I*_*0*_ / *g*_*L*_. Given this constraint we have the parameters {*C*,*g*_*L*_,*V*_*T*_,*V*_*reset*_,Δ_*T*_,*τ*_*w*_,*a*,*b*,*a*_*E*_} for the conductance-based model.

For both the current-based and conductance-based inputs we then optimize model parameters to match the observed voltage fluctuations and spike timing using derivative-free search (Nelder-Mead) with random restarts. Specifically we aim to minimize a cost function that mixes voltage and spike timing terms

$$l=\sum_{t\notin N'}{\left(V(t)-\hat{V}(t)\right)}^{2}+D_{R}(\tau_{R}).$$

Here the first term allows us to minimize the discrepancy between the observed and simulated membrane potential (neglecting voltage changes within 5ms of observed post-synaptic spikes *N*). The second term, the van Rossum distance [90], accounts for discrepancy between observed and simulated spike times, and here we use *τ*_*R*_ = 2 / *f* where *f* denotes the post-synaptic firing rate of the observed neuron. Together these terms allow us to closely approximate the current-injection experiments and simulate model neurons receiving similar current-based or conductance-based input.

## Supporting Information

S1 Fig Spike statistics in observed and simulated neurons. Inter-spike interval distributions and cross-correlograms for synaptic inputs of different strength in a neuron recorded in a slice (“Observed”) along with results from two adaptive-exponential integrate-and-fire simulated neurons, receiving inputs either via current-based or conductance-based synapses. In all three cases, the same presynaptic spike times and weights were used. Model neurons were optimized to fit the observed voltage fluctuations and spike timing.
(EPS)

S2 Fig Parameters and model accuracy for simulated neurons. Parameters and error metrics for the current-based and conductance-based adaptive-exponential integrate-and-fire models fit to two different observed neurons (Layer 2/3 pyramidal cells). Box plots denote median, inter-quartile range and 1.5x inter-quartile range for 60 (s2905) or 61 (s2906) experimental trials with post-synaptic firing rates of 1Hz, 5Hz, and 10Hz (combined). Outliers not shown for clarity. *R* denotes membrane resistance (1 / *g*_*L*_), *τ* denotes the membrane time constant, *DT* determines the strength of the exponential nonlinearity near threshold, while *a*, *b*, and *τ*_*w*_ determine the dynamics of the adaptation variable. For the conductance-based models the scaling factor *A* (*a*_*E*_ in the text) converts the presynaptic conductances to currents.
(EPS)
